# Transfusion Successful in a Case of Post-Partum Hemorrhage

**Published:** 1871-05

**Authors:** 


### *Transfusion Successful in a Case of Post-partum Hemorrhage.*

[From the Dublin Quarterly Journal of Medical Science.]

Dr. Thomas E. Beatty, M.R.I.A., in a graphic report of a case of post-partum hemorrhage in which it was manifest that the patient had not many minutes to live, recommended transfusion. The husband furnished the blood, and about ten ounces were drawn from his arm by Dr. R. McDonnell. This, as it flowed into a common bowl, was agitated briskly with a glass rod, in order to separate the fibrine; and the agitation was kept up after the full quantity had been drawn, and for some minutes after, until a large coagulum of fibrine adhered to the glass rod. During all this time the bowl was kept floating in a basin of hot water. The blood was then strained through a muslin cloth, and all the fibrine was thus separated. All this was done in the parlor, and the blood thus prepared was carried up to the patient's bedroom. The difficulty of the operation now commenced. The patient was as a corpse, bloodless; there was no trace of a vein to be found in her arm. A ligature tied round above the elbow showed nothing. By feeling cautiously, what we imagined to be a vein was perceived. Dr. McDonnell now pinched up a fold of skin at the bend of the arm, ran a narrow bistoury through it, split it up, and displayed the veins at the bottom, resembling small, flat, dead earth-worms. The median basilic appeared to be the largest; this he raised and opened. The bowl of blood, which, during this necessarily tedious proceeding, had been kept floating in hot water, was now brought to the bedside. The injecting apparatus was of the simplest construction. A common brass enema syringe that holds about two ounces, such as is used for self-administration, was fitted with a fine india-rubber tube coming off from the side. This was about a foot long, and terminated in a fine glass tube, four inches long, running to a fine point at its extremity.* I held the bowl containing the blood

*The glass tube was used in order to facilitate the detection of any bubble of air, if such should gain entrance into the apparatus.

with my left hand, and with the right I held the brass syringe standing upright in the blood, and keeping the lower end of it well pressed to the bottom, so as to prevent the possibility of any air gaining access to the interior. Mr. Colles worked the piston slowly and cautiously. The instrument was filled with blood till it ran out of the nozzle, and Dr. McDonnell proceeded to introduce the glass tube into the vein. But this was very difficult to effect; for, although a very sufficient opening had been made into the vein, it was so flat and collapsed that some time elapsed before he could accomplish his object. At length he succeeded, and Mr. Colles urged the blood forward by lowering the piston. About six or seven ounces of blood were thus poured into the system of the patient. It was intensely interesting to watch the effect produced by the introduction of this vital fluid. The first change I noticed was the improvement in respiration: the long, labored, gasping, sighing effort that had been so distressing to witness became more calm and like what natural respiration should be. This began to appear when about half the quantity of blood had been injected. The countenance became less ghastly, and imminent death seemed warded off when the last portion of blood had entered her vein. The restlessness and tossing still continuing, I gave her thirty drops of Batley's solution, and repeated the dose in half an hour; a third dose had the effect of producing sound sleep, from which in six hours she awoke, warm and conscious, with a distinct though very fast pulse. She asked for food, and ate a good breakfast of tea and toast. On the 23d, at twelve o'clock, she had been given beef-tea and brandy twice, and said she was quite well; her countenance was bright and cheerful; pulse one hundred and forty, small. The patient made a good recovery. This case serves to show that there is no such difficulty in safely performing transfusion as we have been led to imagine; but that, when the principles which should govern the operator are fully comprehended, it rests upon his dexterity and skill to carry them out, and not upon a complicated and unwieldly piece of machinery. Like many other highly important operations, it is all in the using, not the costliness of the tools.

